# Development of Technology for Providing Antimicrobial Properties to Medical Disposable Masks

**DOI:** 10.3390/polym16213005

**Published:** 2024-10-26

**Authors:** Kristina Dubinskaitė, Vitalija Rubežienė, Audronė Sankauskaitė, Virginija Skurkytė-Papievienė

**Affiliations:** Department of Textile Technologies, Center for Physical Sciences and Technology, Demokratų Str. 53, LT-48485 Kaunas, Lithuania; vitalija.rubeziene@ftmc.lt (V.R.); audrone.sankauskaite@ftmc.lt (A.S.); virginija.skurkyte@ftmc.lt (V.S.-P.)

**Keywords:** medical masks, antibacterial additives, antimicrobial polymer coating, antibacterial activity, antibacterial efficiency, breathability, microbial purity

## Abstract

Wearing masks to protect against communicable diseases is an effective tool used in many countries affected by the COVID-19 pandemic. The antibacterial activity, antibacterial efficiency, microbial purity, and breathability properties of medical disposable masks are very important. Ag is most commonly applied to antimicrobial textiles. In this work, three antimicrobial additives were used. Four compositions of the binders with antimicrobial additives were prepared and applied to one-layer non-woven PP material. The influence of the binder antimicrobial polymer coating on the breathability and antibacterial activity of the non-woven PP material was evaluated. The results show that the composition of the polyacrylic acid binder had the least effect on their breathability and samples with the silver chloride formulation showed the best antimicrobial response. Based on the microbiological and air permeability results of the samples of the one-layer non-woven material with coating, the samples of two layers and three layers of the medical mask model were prepared. Microbiological studies have shown that a three-layered medical mask model with silver chloride composition in the middle layer, on both sides of the model, has antibacterial efficiency against three pathogens (*E. Coli*, *K. Pneumoniae*, and *S. Aureus*). The performance of this medical mask model has been found to meet the requirements for type I medical masks according to the EN 14863 standard. Studies have shown that the microbial purity of the mask model is CFU/g < 3.

## 1. Introduction

Based on mathematical modelling, an estimated 89 million medical masks are required for the COVID-19 response each month. For examination gloves, that figure goes up to 76 million, while the international demand for goggles stands at 1.6 million per month [1,2]. The Global Smart PPE Market is expected to grow at more than 13.4% CAGR from 2021 to 2031 [3]. Wearing masks to protect against communicable diseases is an effective tool used in many countries affected by the COVID-19 pandemic. The use of respirators and face masks has been shown to significantly reduce the risk of human exposure to beta-coronaviruses (SARS, MERS, and COVID-19) [4]. Scientific research has shown that the virus lives in the environment for a relatively short time: the SARS-CoV2 virus is active at room temperature and 40% relative humidity: on plastic and steel—for about 72 h, on copper—for 4–8 h, on cardboard—for 8–24 h, and 3 h for aerosol particles [5]. The mutations caused by the constant changes in the virus render available drugs and vaccines inactive. Therefore, the wearing of face masks is a rapid and effective protection against infectious diseases caused by airborne pathogens. Face masks are a physical barrier to the inhaled droplets that enter the human body through the nose and mouth, so they can also be used to trap fluids excreted from infected mucous membranes [6,7]. Modern medical masks are usually disposable, made of non-woven polypropylene (PP) of various thicknesses and porosities with a surface density of 20–25 g/cm^2^. They also can be made of polyester, glass fiber, polycarbonate (PC) or polyethylene (PE) fibers [8,9], or natural fibers [10]. The level of filtration of the mask depends on the nature of the fiber used, the method of manufacture, the structure of the filter layer, and the cross-sectional shape of the fiber. Medical disposable face masks usually use three layers of different non-wovens made of spun-bond or melt-blown to filter out contaminants and pathogens of various sizes in the air.

**Figure 1:**
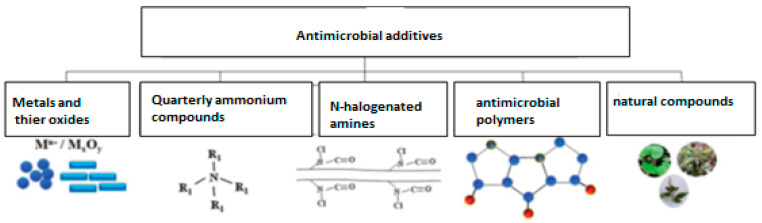
Chemicals giving antimicrobial properties to textiles.

Studies show that microbes on the surface of various materials, including the inner and outer layers of the face mask, can be detected within a period of 4 to 7 days and become a source of transmission of the infection. The moisture accumulated in the capillaries of the mask fiber causes an action by which the trapped microorganisms are transported deeper into the inner layers of the textile and are harmful to human health. Re-aerosolization is possible for a person wearing a mask during intense sneezing or coughing, during which the deposited particles re-enter the body. As the COVID-19 pandemic continues and people continue to wear face masks, there has been an increase in the number of masks worn [11]. One way to reduce the growth of waste medical masks would be to give them antimicrobial properties. This would protect the consumer from microorganisms and secondary infections, prolong the wear time of medical masks, and reduce consumption. The antimicrobial properties of textile face masks can be provided by the introduction of natural and synthetic antimicrobial additives of various chemical compositions (Figure 1).

**Figure 2:**
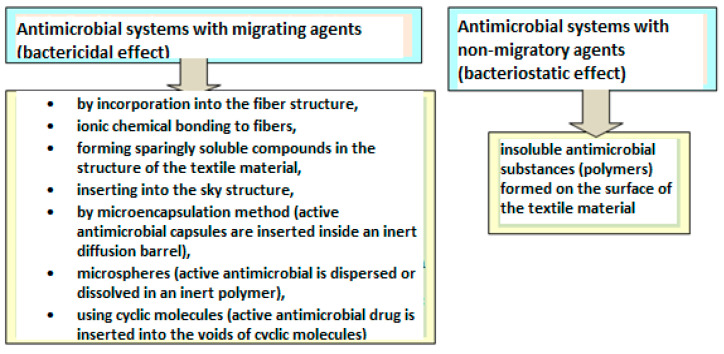
Methods of conferring antimicrobial properties and the mode of action of antimicrobial compounds.

Some metals and their oxides, compounds with quaternary ammonium or phosphonium groups, antimicrobial polymers, N-halamines, peptides, and natural compounds have antimicrobial activity [10,11,12,13,14,15,16,17,18,19]. From time immemorial, Ag and its derivatives have been considered a particularly effective antimicrobial agent. Ag is most commonly applied to antimicrobial textiles, but several studies are known about Ag nanoparticles (AgNPs) embedded directly in face masks [20]. Currently, antimicrobial properties for textiles and PPE, including reusable face masks, are provided by HeiQ Materials AG’s silver (Ag) antimicrobial products HeiQ PURE and antiviral products HeiQ Viroblock [21]. Rudolf GmBH’s RUCO-BAC AGP with silver chloride (AgCl) active ingredient also has antimicrobial activity [22]. Recently, a new generation of bacteriostatic chemicals has been found to form a polymeric protective film that inhibits the growth of microorganisms on textiles. These compounds include Devan Chemicals’ BI-OME AM10. It is an organic silane with active quaternary amino functional groups, which not only bind to the fibers by chemical bonding but also to each other, resulting in a permanent polymeric coating that provides a barrier to microbes on the textile surface [23]. The antimicrobial properties of textiles can be imparted by impregnation, spraying, or the formation of surface coatings with different antimicrobial agents (Figure 2). There is currently a growing focus on nanofiber non-wovens for face masks. Nanofibers with efficient filtration properties are usually prepared by melt-blowing or electrospinning methods. Chemical compounds that provide antimicrobial properties to textiles, as well as antimicrobial fibers, are subject to stringent requirements for toxicity and allergic reactions [24]. Chemical compounds are currently used to form a barrier for microbes, the structure of which allows them to be strongly attached to the fiber.

The aim of the present work was to develop and investigate the polymer composition required for the coating with silver (Ag) nanostructures, which gives the non-woven PP material antimicrobial properties; to investigate the air conductivity of one-layer fabric to intend to choose the particular fabric for a medical mask model; to investigate the antibacterial properties of coated non-woven PP material; to test polymer composition coating technology on non-woven PP material; and to investigate the air conductivity and antimicrobial efficiency of the face mask model used for the production of disposable medical masks.

## 2. Materials and Methods

**Scheme 1:**
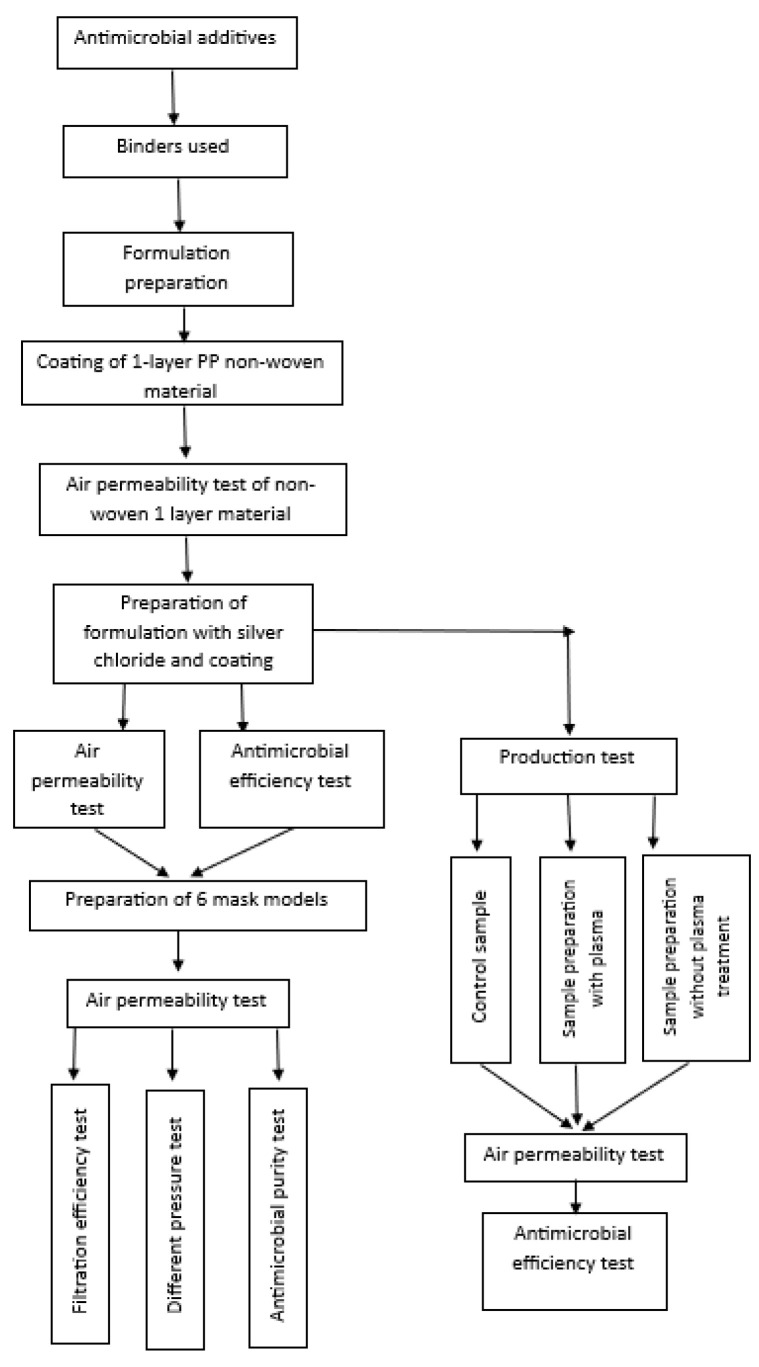
Description of investigation.

This investigation can be described by Scheme 1.

### 2.1. Materials

Three different antibacterial additives (Table 1) were used for providing antimicrobial properties to the selected non-woven fabric intended for use as the inner layer of the disposable face mask.

Different types of binders were used to incorporate antimicrobial additives onto the surface of the textile material (Table 2).

For the preparation of the face mask model, non-woven fabrics were used (Table 3).

### 2.2. Methods

#### 2.2.1. Coating of Non-Woven Fabric Samples with Prepared Compositions

**Figure 3:**
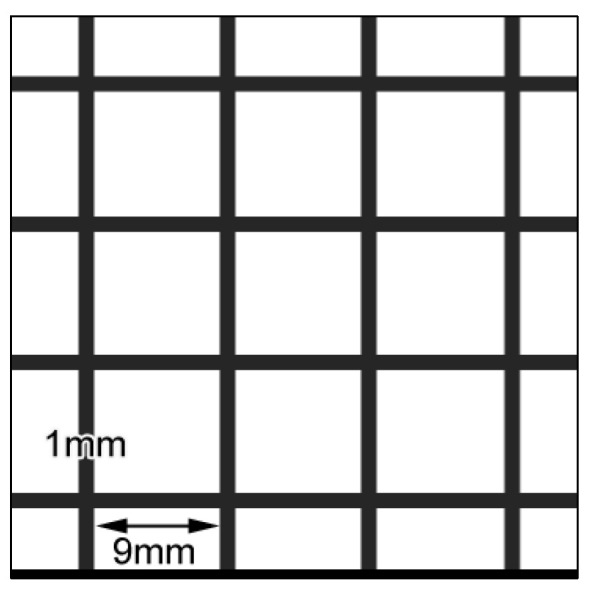
Pattern of fabric partly coated with corresponding composition. In the pictures, black color shows coated places.

**Figure 4:**
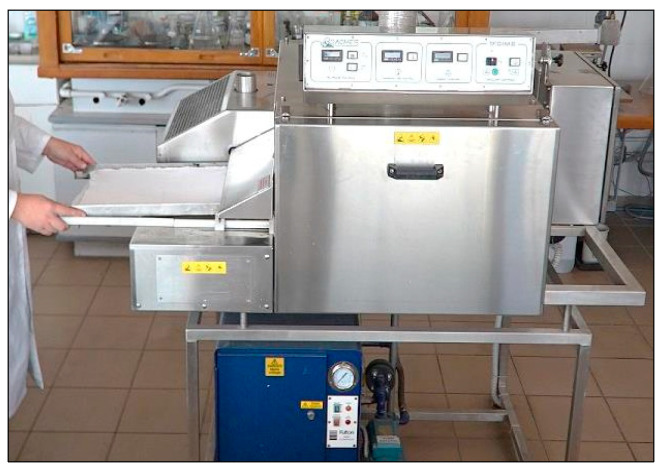
Laboratory oven and steamer TFOS IM 350.

Samples of non-woven fabric primarily were coated with compositions containing different binders (Table 2) using the screen printing method, with a particular squared pattern shown in Figure 3, or fully coating their surface with the corresponding composition. In order to bond and fix the coated layer on the fabric, the samples after screen printing were dried in a laboratory oven and steamer TFOS IM 350 (Figure 4) at a temperature of 100 °C for 3–4 min.

**Figure 5:**
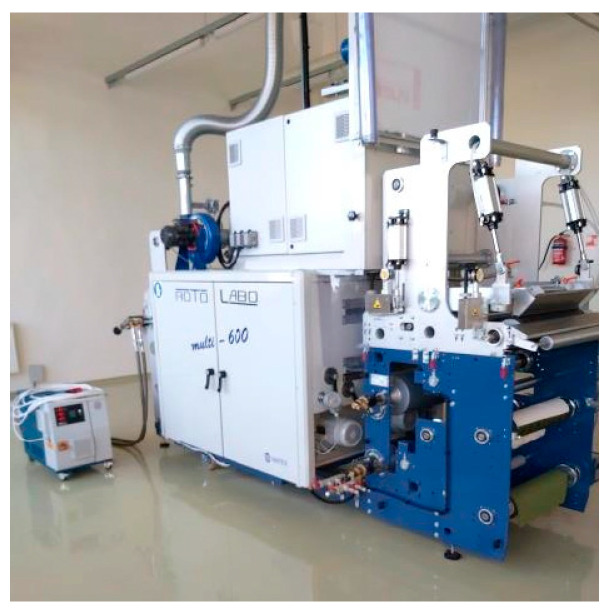
Pilot continuous coating and laminating machine ROTOLAB Multi 600.

**Figure 6:**
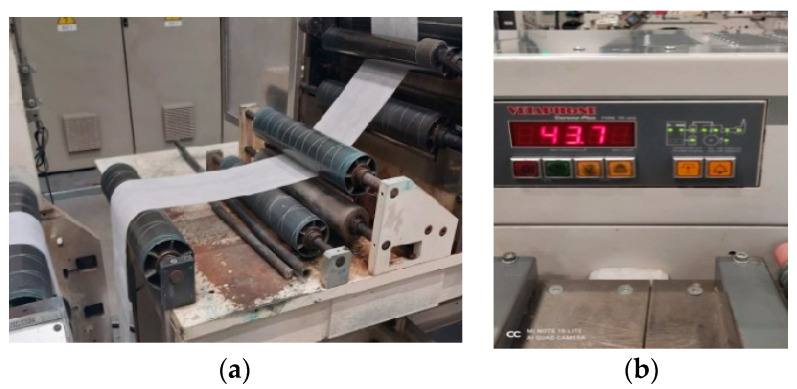
(**a**) OMET packaging printing machine; (**b**) atmospheric discharge plasma section Corona Plus Type TF415.

After the best binder was selected, formulations containing the binder and particular antimicrobial additive (Table 1) were prepared and applied on the material using several coating methods: the same screen printing method as it was used in the case of the binder’s compositions; knife-over-roll coating, using a Rotolab Multi 600 pilot continuous coating and laminating machine (Figure 5); and an industrial OMET packaging printing machine equipped with an atmospheric plasma treatment section (Figure 6).

##### 2.2.2. “Breathability” Test

The “breathability” of the test material was assessed by determining the air permeability (R) according to the EN ISO 9237 standard method [25], which is commonly used for textile fabrics. The device FAP-1034-LP (Frazier Precision Instrument Company, Inc., Hagerstown, MD, USA) was used. The tests were performed at a test area of 20 cm^2^ and a pressure difference of 100 Pa. The samples were conditioned and tested in a standard atmosphere at a temperature of (20 ± 2) °C and a relative humidity of (65 ± 4)%. During the test, the flow rate of the air passing through the specified area of the material is measured at the specified pressure difference.

Another method used to assess the breathability of medical masks that must meet the requirements of EN 14683 [26] is that described in Annex C of EN 14683. According to this method, the differential pressure required to pass a constant flow of air (flow rate 8 L/min) over the measured surface area (4.9 cm^2^) is measured. Document CW 17553 [27] prepared by the European Committee for Standardization shows the correlation between the different breathability parameters of a material:

- The differential pressure of 60 Pa/cm^2^ corresponds to an air permeability of about 93 mm/s at a pressure difference of 100 Pa;
- The differential pressure of 70 Pa/cm^2^ corresponds to an air permeability of about 80 mm/s at a pressure difference of 100 Pa.

#### 2.2.3. Antibacterial Activity Test

The antibacterial activity of the samples of non-woven PP material coated with various antimicrobial coating compositions was determined according to the test method EN ISO 20645. Specimens of the material to be tested are placed on two-layer agar plates. The lower layer consists of a culture medium free of bacteria and the upper layer is inoculated with the selected bacteria. The textiles are tested on both sides. The level of antibacterial activity is assessed by examining the extent of bacterial growth in the contact zone between the agar and the specimen and, if present, the extent of the inhibition zone around the specimen [28].

#### 2.2.4. SEM

Scanning electron microscopy (SEM) images of the surface morphology were obtained using the Quanta 200 FEG device (FEI, Waltham, MA USA).

## 3. Results and Discussion

Four compositions were prepared from the binders listed in Table 2: Tubicoat Thickener-LP—neutralized polyacrylic acid, CHT—Alginat SMT—sodium alignate, Prematex—RA 126—acrylic polymer, and Prisulon—CM—cellulose ether. The compositions of these binders were applied to the non-woven PP (Table 3) in the screen printing method. The samples were then dried in a laboratory dryer—thermostabilizer TFO/S IM 350 at 100 °C for 3–4 min (Figure 4). The labelling of the coated samples is given in Table 4.

The composition of the polyacrylic acid binder polyacrylic acid showed the best air permeability properties, as it had the least effect on their breathability (samples LLP and DLP); therefore, further studies of the antimicrobial coating composition were performed using the binder polyacrylic acid and antimicrobial additives of a different chemical nature and concentration—silver chloride and Nano Ag aqueous dispersion (Table 2). Six formulations of the antimicrobial coating composition with the antimicrobial additives listed in Table 1 were prepared and the antimicrobial efficiency was investigated. Samples with the silver chloride formulation of Formulations B and C (Table 5) showed the best antimicrobial response. Therefore, polyacrylic acid with different concentrations of the silver chloride antimicrobial additive was prepared according to Formulations B and C in Table 5 and applied to the non-woven PP material in two ways: using the screen printing method, with a particular squared pattern (Figure 3), and knife-over-roll coating in the ROTOLAB 600 pilot continuous coating and laminating machine (Figure 6). Textile samples with full coating and partial coating (squared or dot pattern), respectively, were dried at 100 °C in a TFO/S IM350 dryer (Figure 4). The labelling of the coated samples is given in Table 5.

Based on the microbiological and air permeability results, samples were prepared of the one-layer non-woven PP material with coating (Table 6). These samples were used together for one or two layers of the control non-woven material for two and three layers of the medical mask model (Table 6).

Also, the production test was performed in the course of which the white non-woven PP material was coated with silver chloride composition (Formulation C). The composition of the binder composition and the labelling of the non-woven PP-coated binder samples are given in Table 7.

### Production Test

Also, the production test was performed in the course of which the white non-woven PP material was coated with silver chloride composition (Formulation C) in squared pattern coating with a silicone shaft in the packaging printing machine OMET (Figure 6a) without and with treatment in the Corona Plus Type TF415 section (Figure 6b). The speed of the non-woven PP material in OMET was 20 m/min, and the drying temperature was 80 °C. During the production test, 2.5 kg of silver chloride composition (Formulation C) was prepared and applied to the white non-woven PP material.

The following is a description of the production samples:

1. Non-woven PP material (control, KG) was used for the production test in the OMET device;
2. Non-woven PP material coated with a squared pattern drawing coating in the OMET device without Corona discharge treatment;
3. Non-woven PP material coated with a squared pattern drawing coating in the OMET device with Corona discharge treatment.

At the beginning of the work, the influence of the binder of the antimicrobial polymer coating on the breathability of the non-woven PP material was evaluated by measuring the air permeability of the coated material. For this purpose, four compositions were prepared from the binders listed in Table 1: polyacrylic acid, sodium alignate, acrylic polymer, and cellulose ether. The air permeability of the one layer of non-woven PP material with binders of a different chemical nature and coatings of different surface areas formed therefrom was measured according to the standard EN ISO 9237 [25]. The air permeability results presented in Table 8 show that this parameter was influenced not only by the surface area of the formed coating but also by the chemical composition and concentration of the binder. LLP (squared coating) and DLP (full coating) samples had the best air permeability properties of the one layer of non-woven PP fabric with binder coating. The air permeability of the samples of the one layer of non-woven PP material with different chemical binder coatings (Table 8) showed that the composition of the polyacrylic acid binder had the least effect on their breathability (samples LLP and DLP).

Therefore, further studies of the antimicrobial coating composition were performed using the binder polyacrylic acid and antimicrobial additives of a different chemical nature and concentration—silver chloride, organic silane, and Nano Ag aqueous dispersion (Table 1). Six formulations of the antimicrobial coating composition with the antimicrobial additives listed in Table 1 were prepared and their antimicrobial efficiency was investigated. Samples with the silver chloride formulation of Formulations B and C showed the best antimicrobial response.

The antimicrobial properties of non-woven PP-coated samples were evaluated according to the method of the EN ISO 20645: 2005 [28]. The resistance of the coated samples to microorganisms (Escherichia coli, Klebsiella pneumoniae, and Staphylococcus aureus) was evaluated and the effect of antibacterial activity was determined. The effect of antibacterial activity is determined by the growth pattern of the micro-organism/bacterial colony and is defined as “good” (no colony growth), “limited” (low colony growth), and “no effect” (colony growth). Samples of the one layer with antimicrobial coating and multilayer packages consisting of two or three layers of the non-woven material, one of which was coated with antimicrobial coating, were examined. The obtained antibacterial activity test results are presented in Table 9, respectively.

At the beginning of the study, the air permeability tests of the one, two, and three layers of the control sample containing uncoated non-woven PP material were performed according to the method of EN ISO 9237: 1997 [25]. The obtained results are presented in Table 10.

In parallel, all samples of the one layer of non-woven PP material with antimicrobial coatings prepared according to Formulations B and C, respectively, were tested for air permeability, and the results are shown in Table 11.

Based on the microbiological and air permeability results of the samples of the one layer of non-woven PP material with coating, a sample with the best antibacterial effect and air permeability was selected, which was used together for the one or two layers of the control non-woven material for the two and three layers of the medical mask models. The antibacterial activity on both sides of the model was evaluated (Table 5), and its air permeability is presented in Table 12.

**Figure 7:**
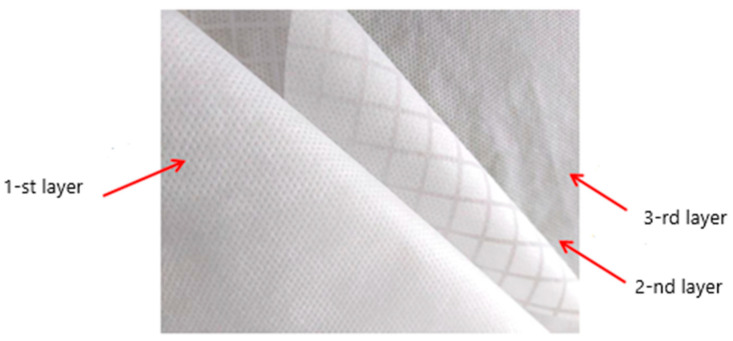
The arrangement of 1st, 2nd, and 3rd layers of non-woven PP material in layout M 890.

Microbiological studies of the one layer of non-woven PP material with antimicrobial coating showed good antibacterial activity against all three pathogens: *E. Coli*, *K. Pneumoniae*, and *S. Aureus*. MA208499 (Formulation C), MA21466 (Formulation C), and MA892 (Formulation B) refer to the samples coated with silver chloride composition, respectively, with a complete and partial coating of the material surface by a squared pattern or dots pattern. The samples coated with other antimicrobial additives had limited/insufficient or selective antibacterial activity. When analysing the air permeability results of the one-layer samples with antimicrobial coating (Table 11), it was found that the worst breathability—80 mm/s at a pressure difference of 100 Pa—was characterized by sample MA20849 with a continuous coating of silver chloride composition (Formulation C). By summarizing the air permeability of the one-layer samples (Table 10) and the antibacterial efficiency results (Table 9), it can be stated that good antibacterial activity was observed for all three pathogens (Escherichia coli, Klebsiella pneumoniae, and Staphylococcus aureus) and the air permeability (341 mm/s) of sample MA21466 with squared pattern coating of silver chloride composition (Formulation C). Based on the obtained research data, a three-layer medical mask model MA890 was prepared, with the first and third layers consisting of raw non-woven PP material (Table 3), and the second layer consisting of sample MA21466 (Table 6); the air permeability of the M 890 model (Table 12) and the antibacterial activity of both sides (Table 9) were investigated. The arrangement of the non-woven PP material layers in the M 890 layout is shown in Figure 7.

**Figure 8:**
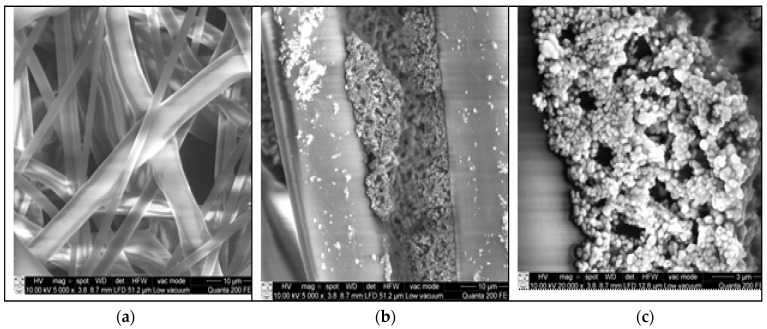
Mask model MA890 2nd layer with RUCO-BAC AGP composition coating SEM image: (**a**) PP fiber without coating, magnification 5000×, (**b**) AgCI attached to PP fiber particles, magnification 10,000×; and (**c**) AgCI particles attached to the fiber, magnification 20,000×.

The air permeability of the three-layer medical mask model MA890 measured by the method of EN ISO 9237: 1997 was (140 ± 16) mm/s (Table 12). Scanning electron microscopy (SEM) of the surface morphology of the second layer of the mask model with the squared pattern coating of silver chloride composition (Formulation C) was performed. SEM images (Figure 8a–c) show AgCl particles accumulated on the fiber strands of the second layer of non-woven PP material (Figure 8b,c). Microbiological studies of the three-layer medical mask model MA890 [28] showed good antibacterial efficiency on both sides for all three pathogens (Table 9). Sample MA890, with a plot of squares in the second layer of silver chloride composition (Formulation C) in which the antimicrobial coating coverage deposit was 5.6 g/m^2^, was found to have good antibacterial efficiency on both sides of the mask for the three pathogens tested (*Escherichia coli*, *Klebsiella pneumoniae*, and *Staphylococcus aureus*).

The performance requirements for medical masks are specified in the standard EN 14683 [26]. According to this standard, medical masks are divided into three types according to their purpose:

- Type I—minimum protection. This type of medical mask is intended to be worn by patients and others to reduce the risk of spreading infections, especially in the event of an epidemic or pandemic;
- Type II—this type of mask is primarily intended for use by healthcare professionals in an operating or other medical environment with similar requirements;
- Type IIR—used when the wearer seeks to protect himself from splashes of potentially contaminated liquids.

Using the EN 14683 standard for medical masks [26], three layers of non-woven PP material (Table 8) were tested for their performance in an M 890 medical mask model, one layer of which was coated with silver chloride antimicrobial composition (Formulation C) coating.

Samples of the M 890 model were named “3-layer material with antimicrobial finish “CUST 01/3” and the following properties were tested: bacterial filtration efficiency (BFE, %), differential pressure (Pa/cm^2^), and microbial purity (CFU/g).

The value of the filtration efficiency of the M 890 model bacteria with the measurement uncertainty was determined to be the following: BFE = (94 ± 2.4)%. According to the requirements of the standard EN 14683 [26], the bacterial filtration efficiency (BFE) must be the following:

- BFE of type I medical masks—≥ 95%;
- BFE of type II and IIR medical masks—≥ 98%.

Based on the results of the study, it can be stated that the BFE value of the mask model with the measurement uncertainty falls within the scope of compliance with the requirements for type I medical masks. Other authors determined that a higher BFE represents a higher level of protection against pathogen transmission [9,15,17].

The differential pressure value of the M 890 model with the measurement uncertainty was determined to be ΔP = (40.9 ± 3.7) Pa/cm^2^. Requirements of the EN 14683 [16] standard include the following:

- The differential pressure of type I and type II medical masks must be <40 Pa/cm^2^;
- Type IIR—<60 Pa/cm^2^.

Therefore, based on the results of the study, it can be stated that the mask model ΔP fully meets the requirements for type IIR masks, and the obtained value with measurement uncertainty falls within the scope of requirements for type I and type II medical masks.

Microbial cleanliness means the absence of a population of viable micro-organisms on a product and/or packaging [26]. Prior to testing, samples were heated at 100 °C for 1 h and then placed in individual polyethylene packages. Studies have shown that the microbial purity of the M 890 model is CFU/g < 3. CFU is a microbial colony-forming unit that expresses the number of microorganisms grown. According to the requirements of the standard EN 14683 [26], the microbial cleanliness (CFU/g) of type I, II, and IIR medical masks must be ≤30; therefore, based on the results of the study, it can be stated that the microbial cleanliness of the mask model meets the requirements of all three types of medical masks [9,10,11,12,13,14,15,16,17,18].

There were two production tests where the white non-woven PP material provided by the company was coated with silver chloride composition (Formulation C) in squared pattern coating with a silicone shaft (Figure 7) in the packaging printing machine OMET (Figure 7) without and with treatment in the Corona Plus Type TF415 section (Figure 7). The speed of the non-woven PP material in OMET was 20 m/min, and the drying temperature was 80 °C. Microbiological studies [28] showed that the antimicrobial squared pattern coating (Formulation C) formed on non-woven during the production test has limited antibacterial efficiency against the three pathogens tested (Escherichia coli, Klebsiella pneumoniae, and Staphylococcus aureus). A comparison of the laboratory and production test results in Table 9 shows that the sieve template had a significantly higher coverage deposit of silver chloride composition (Formulation C) (5.6 g/m^2^) than the OMET device (≤1 g/m^2^) with or without the unloading of CORONA discharge pre-treatment. In both the laboratory and production tests, the air permeability of the non-woven material decreased slightly or not at all (Table 13), respectively. We believe that during the manufacturing test, due to the technological capabilities of the silicone shaft with an engraved 1 cm × 1 cm squared pattern installed in the OMET device, a very small amount of antimicrobial composition was introduced into the textile material, which is insufficient for an effective antimicrobial response. Therefore, we recommend increasing the area of the antimicrobial coating on the textile surface by applying a “reverse” pattern to the squares or by covering the entire surface of the material with a continuous coating. We believe that after each change in the coating pattern, it is necessary to re-test the air permeability to determine the antibacterial efficiency of the coated material. Also, by incorporating functional agents (antimicrobial agents) on masks, superhydrophobic surface treatment may be applied to avoid pathogen adhesion and allow mask self-cleaning [29]. It was determined by other authors that the incorporation of antimicrobial agents achieves a self-disinfectant feature [19]. Even if antimicrobial agents, for example, metal nanoparticles (CuNPs, ANPs, ZnNPs), have low stability and non-adhesion or leaching, they have very good antibacterial activity and do not change air permeability [19], and screen printing or coating does not influence metal leaching [30].

## 4. Conclusions

1. The air permeability results presented in Table 8 show that this parameter was influenced not only by the surface area of the formed coating but also by the chemical composition and concentration of the binder. The LLP (squared pattern coating) and DLP (full coating) samples had the best air permeability properties of the one-layer non-woven PP fabric with binder coating. The air permeability of the samples of the one-layer non-woven PP material with different chemical binder coatings (Table 8) showed that the composition of the polyacrylic acid binder polyacrylic acid had the least effect on their “breathability” (samples LLP and DLP).
2. When summarizing the air permeability of the one-layer samples (Table 8) and the antibacterial efficiency results (Table 9), it can be stated that good antibacterial activity was observed for all three pathogens (*Escherichia coli*, *Klebsiella pneumoniae,* and *Staphylococcus aureus*) and the air permeability (341 mm/s) of sample MA21466 with squared pattern coating of silver chloride composition (Formulation C). Based on the obtained research data, a three-layer medical mask model MA890 was prepared, with the first and third layers consisting of raw non-woven PP material (Table 3) and the second layer consisting of sample MA21466 (Table 6).
3. Microbiological studies have shown that the non-woven PP material (Bermed) of the three-layer medical mask model MA890 with the 5.6 g/m^2^ coverage deposit coating of the antimicrobial silver chloride composition in the middle layer (Formulation C) on both sides of the model has antibacterial efficiency against three pathogens (*E. coli*, *K. pneumoniae*, and *S. aureus*).
4. The performance of the mask model MA890 has been found to meet the requirements for type I medical masks according to the EN 14683 standard [26].
5. This study has shown that the microbial purity of the M 890 model is CFU/g < 3. It can be stated that the microbial cleanliness of the mask model meets the requirements of all three types of medical masks.
6. After the production test in OMET device preparation and application on non-woven PP material for masks’ silver chloride composition (Formulation C), it was determined that in order to achieve good antimicrobial efficiency, it is necessary to modify the existing PTO drawing, increasing the surface area of the textile coating and a coverage deposit.

## Figures and Tables

**Table 1:** Antimicrobial additives used.

Name	Producer	Parameters
*RUCO-BAC AGP*	Rudolf Group	Chemical content (to 1% ≤ AgCl < 2.5%), etc., non-ionic, pH7.
*BI-OME AM10*	Devan Chemicals	Chemical content—organic silane (6–10%), etc., cationic, pH4.
Dispersion of elemental nanosilver (Ag)	NANOIRON	Chemical content—Ag, water, organic stabilizers; Ag concentration—100 ppm; Particle shape—spherical; Average particle size—20–40 nm; pH 8–9, at a concentration of 10 ppm.

**Table 2:** Binders used.

Name of Binder	Producer	Parameters	Content of the Binder Composition Used for Coating, %
*Tubicoat Thickener LP*	CHT R. Beitlich GmbH	Chemical content—polyacrylic acid, anionic, pH 7.5–8.2 (2%).	Binder, 3 Deionized water, 97
*CHT-Alginat SMT*	CHT R. Beitlich GmbH	Chemical content—sodium alginate, anionic, 6.5–8.5 (4%).	Binder, 4 Deionized water-96
*Prematex RA 126*	CHT R. Beitlich GmbH	Chemical content—acrylic polymer (≥1–<2.5), etc., anionic, pH 7–9.	Binder, 20 Deionized water-80
*Prisulon CM70*	CHT R. Beitlich GmbH	Chemical content—cellulose ether, non-ionic, pH 6.5–8.5 (6%).	Binder, 20 Deionized water-80

**Table 3:** Non-woven materials used.

	Parameters of Fabric
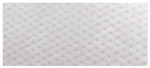	Colour—white; mass per unit area—24.12 g/cm^2^; fiber content—100% polypropylene (PP); produced using *melt-blow method.*

**Table 4:** Labeling and coating composition of non-woven PP with binder coating using screen printing method.

Marking of Samples		Binder Coating Composition, g
Full Coating	Squared Pattern Coating (1 cm × 1 cm × 0.9 cm)	
DLP	LLP	Silver chloride*-3* Deionized water-97
DSMT	LSMT	Sodium alignate*-4* Deionized water-96
D126	L126	Acrylic polymer*-20* Deionized water-80
DCM	LCM	Cellulose ether*-6* Deionized water-80

**Table 5:** The antimicrobial formulation and labelling of coated samples.

Antimicrobial Additive	Formulation, g/mL	Marking of the Sample with Coating		
		Full Coating	Cell Coating	Dot Coating
Silver chloride	Formulation B 1. Silver chloride-30 2. Polyacrylic acid-3 3. Deionized water-67	-	MA21466 MA889 MA891	MA888 MA892
	Total: 100			
	Formulation C 1. Silver chloride-80 2. Polyacrylic acid-3 3. Deionized water-17	MA20849	MA21466 MA890	-
	Total: 100			

- Samples with full coating were not prepared for Formulation B.

**Table 6:** Mask models.

Marking of Sample	Description of Sample Layers
MA888	1st layer: non-woven PP fabric 2nd layer: non-woven PP fabric with dot coating, Formulation B (Table 5)
MA888 + 1 layer	1st layer: non-woven PP fabric 2nd layer: non-woven PP fabric with dot coating, Formulation B (Table 5) 3rd layer: non-woven PP fabric
MA889	1st layer: non-woven PP fabric with squared pattern coating, Formulation B (Table 5) 2nd layer: non-woven PP fabric
MA889 + 1 layer	1st layer: non-woven PP fabric 2nd layer: non-woven PP fabric with squared pattern coating, Formulation B (Table 5) 3rd layer: non-woven PP fabric
MA890 (mask layout)	1st layer: non-woven PP fabric 2nd layer: non-woven PP fabric with squared pattern coating, Formulation C (Table 5) 3rd layer: non-woven PP fabric

**Table 7:** Parameters of non-woven PP materials tested.

Description of the Sample	Mass Per Unit Area g/m^2^	Coverage Deposit, g/m^2^
Non-woven PP material coated with cell pattern silver chloride composition (Formulation C) coating without Corona discharge in *OMET* device	24.4	*Very low* *(≤1)*
Non-woven PP material coated with cell pattern *by* silver chloride composition (Formulation C) coating with Corona discharge in *OMET* device	24.4	*Very low* *(≤1)*
Non-woven PP material (control, used for production test, KG)	25.1	-

- control sample was without coating.

**Table 8:** Air permeability results of one layer of the non-woven PP material with binder coating.

No.	Marking of Samples	Description of Coating	Air Permeability (R), mm/s
1.	L126	Squared pattern coating	348 ± 25
2.	D126	Full coating	84 ± 9
3.	LLP	Squared pattern coating	434 ± 30
4.	DLP	Full coating	369 ± 27
5.	LSMT	Squared pattern coating	376 ± 28
6.	DSMT	Full coating	116 ± 15
7.	LCM	Squared pattern coating	330 ± 24
8.	DCM	Full coating	20 ± 3

**Table 9:** Parameters of antimicrobial properties of one and two layers of non-woven PP material and three layers of the mask model.

Marking of Sample		Antibacterial Activity		
		*E. coli* *ATCC 25922*	*S. aureus* *ATCC 6338*	*K. pneumonia* *ATCC 13882*
MA20849 (1 layer)		*Good*	*Good*	*Good*
MA21466 (1 layer)		*Good*	*Good*	*Good*
MA13915 (1 layer)		*Good*	*Limited*	*Limited*
MA888 (2 layers)	1	*Good*	*Good*	*Good*
	2	*Good*	*Limited*	*Limited*
MA889 (2 layers)	1	*Limited*	*Good*	*Limited*
	2	*Limited*	*Good*	*Limited*
MA890 (3 layers mask model)	1	*Good*	*Good*	*Good*
	3	*Good*	*Good*	*Good*
MA891 (1 layer)		*Good*	*Good*	*Limited*
MA892 (1 layer)		*Good*	*Good*	*Good*

**Table 10:** Air permeability of uncoated non-woven PP material.

Description of the Sample	Air Permeability (R), mm/s
Non-woven PP material, 1 layer	451 ± 22
Non-woven PP material, 2 layers	223 ± 16
Non-woven PP material, 3 layers	150 ± 11

**Table 11:** Air permeability of a single layer of non-woven PP material with antimicrobial coating.

Marking of Sample	Description of the Antimicrobial Coating	Air Permeability (R), mm/s
Control sample, K	Uncoated non-woven PP material	451 ± 30
MA20849	Full coating, Formulation C	80 ± 7
MA21466	Squared pattern coating, Formulation C	369 ± 25
MA891	Squared pattern coating, Formulation B	362 ± 25
MA892	Dot coating, Formulation B	246 ± 26

**Table 12:** Air permeability of the multilayer non-woven material (package) with antimicrobial coating.

Marking of Sample	Description of Sample Layers	Air Permeability (R), mm/s
MA888	1st layer: non-woven PP fabric 2nd layer: non-woven PP fabric with dot coating, Formulation B (Table 5)	185 ± 19
MA888 + 1 layer	1st layer: non-woven PP fabric 2nd layer: non-woven PP fabric with dot coating, Formulation B (Table 5) 3rd layer: non-woven PP fabric	119 ± 15
MA889	1st layer: non-woven PP fabric with squared pattern coating, Formulation B (Table 5) 2nd layer: non-woven PP fabric	203 ± 21
MA889 + 1 layer	1st layer: non-woven PP fabric 2nd layer: non-woven PP fabric with squared pattern coating, Formulation B (Table 5) 3rd layer: non-woven PP fabric	144 ± 16
MA890 (mask model)	1st layer: non-woven PP fabric 2nd layer: non-woven PP fabric with squared pattern coating, Formulation C (Table 5) 3rd layer: non-woven PP fabric	140 ± 16

**Table 13:** Parameters of non-woven PP materials tested.

Description of the Sample	Mass Per Unit Area g/m^2^	Coverage Deposit, g/m^2^	Air Permeability R, mm/s
Non-woven PP material (control used for laboratory tests, K)	24.13	-	451 ± 22
Non-woven PP material coated with cell pattern *by* silver chloride composition (Formulation C) coating without *Corona* discharge in *OMET* device	24.4	*Very low* *(≤1)*	419 ± 28
Non-woven PP material coated with cell pattern *by* silver chloride composition (Formulation C) coating with *Corona* discharge in *OMET* device	24.4	*Very low* *(≤1)*	452 ± 22
Non-woven PP material (control, used for production test, KG)	25.1	-	445 ± 28
Non-woven PP material coated with sieve template by silver chloride composition (Formulation C) with cell pattern coating	29.73	5.6	369 ± 18

- control sample was not coated; therefore, coverage deposit was not measured.

## Data Availability

The original contributions presented in the study are included in the article, further inquiries can be directed to the corresponding author.
